# Mechanistic study on the reduction of TNF-α and β-CTX levels in RA patients by moxibustion combined with western medication through regulation of the Wnt/β-catenin pathway

**DOI:** 10.3389/fimmu.2026.1808931

**Published:** 2026-05-22

**Authors:** Hui Wang, Heshan Chen, Yuan Li, Yi Yang, Xiaojuan Hong, Ping Wu

**Affiliations:** 1 College of Acupuncture and Tuina, Chengdu University of Traditional Chinese Medicine, Chengdu, Sichuan, China; 2 Department of Rheumatology, Chengdu University of Traditional Chinese Medicine Affiliated Hospital, Chengdu, Sichuan, China

**Keywords:** bone metabolism, moxibustion, rheumatoid arthritis, TNF- α, Wnt/β -catenin signaling pathway, β -CTX

## Abstract

**Background and aims:**

The pathological core of rheumatoid arthritis (RA) is progressive bone destruction driven by chronic inflammation. The Wnt/β-catenin signaling pathway plays a key regulatory role in bone metabolism, but it is disrupted by the inflammatory environment in RA. This study investigates the mechanism by which moxibustion combined with conventional Western medicine modulates the Wnt/β-catenin signaling pathway to coordinate inflammation and bone metabolism in rheumatoid arthritis (RA).

**Methods:**

This study randomly assigned 70 patients to a moxibustion group (n=35) and a control group (n=35). The control group received oral methotrexate (10 mg/week) combined with folic acid (10 mg/week). The moxibustion group received the same medication as the control group plus moxibustion therapy primarily targeting Zusanli (ST36), and Ashi points, administered three times weekly for a 4-week course. Clinical symptoms and laboratory parameters were compared between groups, and serum levels of tumor necrosis factor-alpha (TNF-α), interleukin-17A (IL-17A), wingless-type MMTV integration site family member 3A (WNT3A), low-density lipoprotein receptor-related protein 6 (LRP-6), glycogen synthase kinase-3 beta (GSK-3β), β-catenin, osteoprotegerin (OPG), and beta-C-terminal telopeptide of type I collagen (β-CTX) were measured.

**Results:**

Compared with the conventional control group alone, moxibustion combined therapy significantly improved clinical symptoms in RA patients (P<0.05). Mechanistically, the moxibustion group simultaneously downregulated serum proinflammatory factors (TNF-α, IL-17A) and bone resorption marker β-CTX, suppressed abnormally activated Wnt pathway molecules (WNT3A, LRP-6, β-catenin), and upregulated the bone protective factor OPG while inhibiting GSK-3β (P < 0.05 for both intra- and intergroup comparisons). Correlation analysis revealed positive correlations between β-catenin changes and those in TNF-α and WNT3A (*P* < 0.05).

**Conclusion:**

The combination of moxibustion and Western medicine effectively alleviates clinical symptoms in patients with rheumatoid arthritis and suggests a potential to delay structural damage. Its mechanism involves suppressing key inflammatory mediators TNF-α and IL-17A, thereby activating and regulating the Wnt/β-catenin signaling pathway. This modulates serum levels of OPG and β-CTX, leading to systemic improvement in bone metabolic balance and exerting a multi-target therapeutic effect.

## 1 Introduction

Rheumatoid arthritis (RA) is an autoimmune disease characterized by chronic synovial inflammation and progressive destruction of bone and cartilage (1). Its bone destruction stems not only from excessive osteoclast activation but also from insufficient bone formation due to impaired osteoblast function. Although modern medical treatments, represented by disease-modifying antirheumatic drugs (DMARDs) such as methotrexate, effectively control disease progression in most patients, some individuals still face challenges including suboptimal efficacy, drug side effects, and joint function decline due to prolonged disease activity (2, 3). Therefore, exploring safe and effective complementary or alternative therapies to synergistically improve clinical symptoms and delay structural damage represents a key direction in current RA clinical management. The Wnt/β-catenin signaling pathway plays a central role in regulating osteoblast differentiation and bone formation. However, within the inflammatory environment of RA, this pathway is suppressed by pro-inflammatory factors such as tumor necrosis factor-alpha (TNF-α) and interleukin-17A (IL-17A) (4, 6). Concurrently, abnormal activation of its downstream effector molecules (such as β-catenin) in synovial cells exacerbates inflammation and destruction, creating a vicious cycle of “inflammation-induced bone destruction.”

Moxibustion, as an outstanding representative of external therapies in traditional Chinese medicine, possesses the efficacy of warming meridians, unblocking channels, dispelling cold, removing dampness, and harmonizing qi and blood (5, 7). Extensive clinical observations indicate that moxibustion can effectively alleviate joint swelling and pain as well as morning stiffness in RA patients, thereby improving their quality of life (1, 8). WNT3A is a prototypical canonical Wnt ligand that activates this pathway by binding to the Frizzled receptor and LRP-5/6 co-receptor, leading to β-catenin stabilization and nuclear translocation. In bone metabolism, WNT3A promotes osteoblast differentiation and bone formation, and its dysregulation has been implicated in the pathogenesis of rheumatoid arthritis-associated bone erosion (38, 39).However, moxibustion’s specific biological mechanisms remain incompletely elucidated, which to some extent limits its standardized application and promotion within modern comprehensive treatment regimens. Recent studies indicate that the Wnt/β-catenin signaling pathway plays a complex and pivotal role in the pathogenesis of RA (9, 27). It not only regulates the abnormal activation and proliferation of synovial fibroblasts but also forms an interactive network with inflammatory mediators such as TNF-α and IL-17A, collectively influencing osteoclast differentiation and bone metabolic balance. The abnormal activation of this pathway is considered a key molecular basis for RA-related bone erosion.

Based on this, the study hypothesizes that the clinical benefits of moxibustion therapy for RA may be related to its regulation of the Wnt/β-catenin signaling pathway, thereby influencing downstream inflammatory responses and bone metabolic balance. To validate this hypothesis, we designed and conducted a randomized controlled trial. This study aims to: 1) Objectively evaluate the clinical efficacy of combining moxibustion with conventional Western medication for RA; 2) Detect the effects of moxibustion on levels of key inflammatory cytokines, Wnt/β-catenin pathway-related proteins, and bone metabolism markers in patient serum; 3) Preliminary explore the association between clinical symptom improvement and the aforementioned molecular changes, thereby providing new experimental evidence to elucidate the scientific basis of moxibustion therapy for RA.

## 2 Clinical data

### 2.1 Patient sources

All subjects in this study were recruited from the outpatient department of the Rheumatology and Immunology Division at Sichuan Provincial Hospital of Traditional Chinese Medicine between March 2024 and March 2025. Included patients met the RA diagnostic criteria and trial inclusion criteria outlined below, voluntarily participated in the study, and signed written informed consent forms.

### 2.2 Diagnostic criteria

The diagnosis of RA is based on the criteria jointly established by the American College of Rheumatology (ACR) and the European League Against Rheumatism (EULAR) in 2010 (10) (see **Table 1** for details):

**Table 1:** 2010 ACR/EULAR diagnostic criteria for rheumatoid arthritis.

Project		Rating
A. Joint involvement	one large joint	0
	2 to 10 major joints	1
	1–3 small joints affected (with or without involvement of large joints)	2
	4–10 small joints affected (with or without involvement of large joints)	3
	>10 joints (including at least 1 small joint)	5
B. Serum	RF and anti-CCP antibodies are both negative.	0
	At least one low-titer positive result for RF or anti-CCP	2
	At least one high-titer positive result for RF or anti-CCP	3
C. Acute Phase Reactants	CRP and ESR are both within normal limits.	0
	Elevated CRP or ESR	1
D. Duration of Synovitis	<6 weeks	0
	≥6 weeks	1

1. Definition of joint involvement: Refers to joints exhibiting clinical tenderness and swelling, excluding the distal interphalangeal joints, first carpometacarpal joints, and first metatarsophalangeal joints during assessment. Radiographic evidence of erosive changes may also serve as a basis. 2. Joint classification: Large joints: Shoulder, elbow, hip, knee, ankle joints. Small joints: Metacarpophalangeal joints, proximal interphalangeal joints, thumb interphalangeal joints, wrist joints, and second to fifth metatarsophalangeal joints.

Definition of serological positivity: High-titer positive: Test value > 3 times the upper limit of normal. Low-titer positive: Test value > upper limit of normal but ≤ 3 times the upper limit of normal.

Diagnostic criteria: A cumulative score of ≥6 points across items A-D, with at least one positive result in either item B or C, is required for a confirmed diagnosis of rheumatoid arthritis (RA). RF, rheumatoid factor; anti-CCP, anti-cyclic citrullinated peptide antibody; CRP, C-reactive protein; ESR, erythrocyte sedimentation rate.

### 2.3 Inclusion criteria

(1) Patients meeting diagnostic criteria for rheumatoid arthritis;
(2) Patients aged 18 years or older, male or female;
(3) Disease Activity Score 28 (DAS28) (11) ≥ 3.2 points;
(4) Soft tissue swelling or effusion in at least 3 joints;
(5) Patients must be conscious and able to cooperate with the study;
(6) Not currently participating in any other ongoing clinical studies;
(7) Voluntary participation in this study and signing of an informed consent form.

### 2.4 Exclusion criteria

(1) Does not meet the 2010 American College of Rheumatology (ACR)/European League Against Rheumatism (EULAR) joint criteria for rheumatoid arthritis (RA) diagnosis;
(2) Concurrent autoimmune diseases: such as systemic lupus erythematosus, ankylosing spondylitis, mixed connective tissue disease, etc.;
(3) Presence of severe concomitant diseases in other systems, including cardiovascular, respiratory, endocrine, hematologic, or severe hepatic/renal disease;
(4) Presence of malignancy;
(5) Pregnant or lactating women, or individuals with psychiatric conditions preventing study compliance;
(6) Concurrent infectious diseases: acute phase of any infection;
(7) Known allergic constitution or documented history of allergy to multiple medications (including study-related drugs);
(8) Use of medications known to affect bone metabolism (including but not limited to bisphosphonates, denosumab, teriparatide, systemic glucocorticoids at a dose equivalent to prednisone >5 mg/day, or biologic DMARDs) within 3 months prior to enrollment.

Note: Participants meeting any of the above criteria are ineligible for inclusion in this study.

## 3 Research subjects and methods

### 3.1 Research design

The sample size was calculated based on data from a similar clinical mechanism study (12). We assumed a clinically meaningful difference in the primary outcome (DAS28 score) of 0.5 between groups, with a standard deviation of 0.6. Using a two-sided significance level (α = 0.05) and a power of 80% (1-β = 0.80), the required sample size was 29 patients per group. To meet the minimum sample size requirement for small-sample clinical studies (n ≥ 30 per group) and to account for a 15% anticipated dropout rate, we planned to enroll 35 patients per group (70 in total). The 70 RA patients were randomly divided into two groups (35 patients each in the moxibustion and control groups). Ultimately, 65 patients completed the study: 32 in the moxibustion group (3 dropouts, 8.57% dropout rate) and 33 in the control group (2 dropouts, 5.71% dropout rate). The actual sample size met statistical requirements; the difference in dropout rates between groups was not statistically significant (P>0.05).

**Figure 1:**
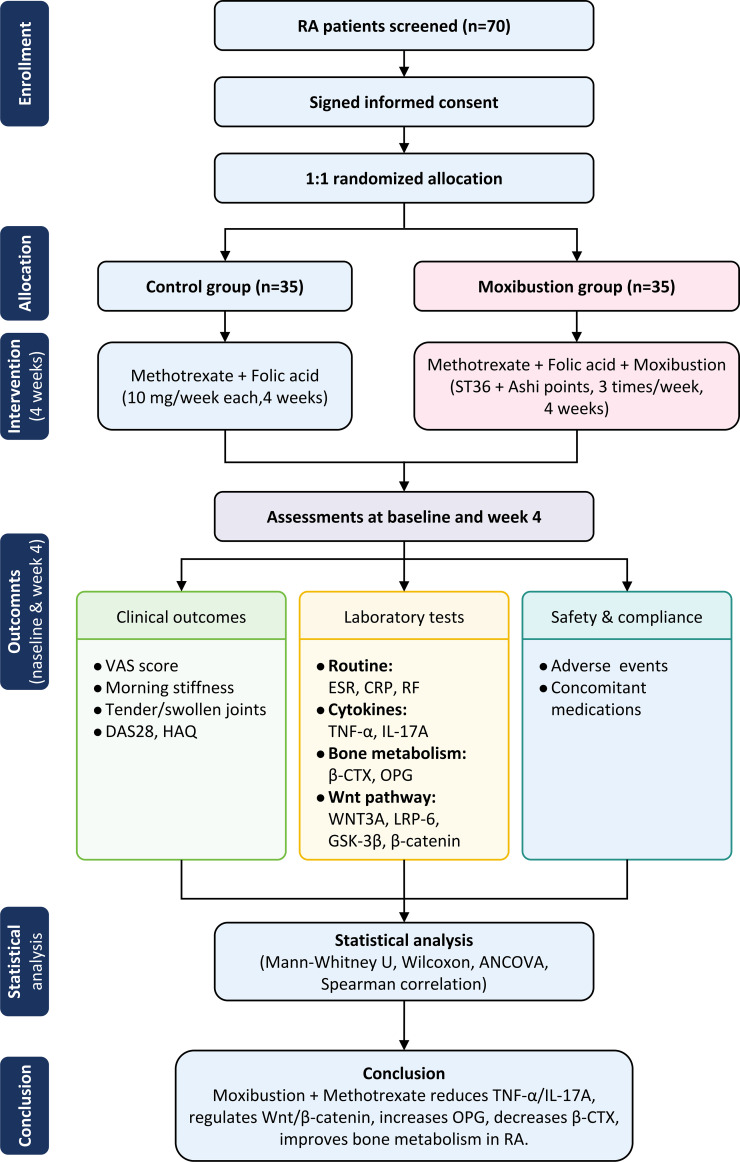
Study design flowchart.

Eligible patients were randomly assigned in a 1:1 ratio to the moxibustion group or the control group using a computer-generated random number table. Allocation concealment was ensured by using sequentially numbered, opaque, sealed envelopes prepared by an independent statistician who was not involved in patient recruitment or treatment. Due to the nature of moxibustion therapy, it was not feasible to blind the patients or the acupuncturists performing the moxibustion. However, to minimize detection bias, outcome assessors (who performed clinical evaluations and laboratory measurements) and data analysts were kept blind to group allocation throughout the study. All analyses were performed on the per-protocol set (patients who completed the full treatment). This study employed a parallel-group design with both between-group comparisons and within-group longitudinal comparisons. Specifically, horizontal comparisons were made between the moxibustion and control groups, while longitudinal comparisons assessed changes in indicators before and after treatment within each group. Refer to **Figure 1** for the flowchart.

### 3.2 Intervention measures

This randomized controlled trial enrolled 70 patients with rheumatoid arthritis (RA), randomly assigned in a 1:1 ratio to two groups: Control group: Oral methotrexate (10 mg/week) + folic acid (10 mg/week), administered weekly for 4 weeks. All medications were taken strictly as prescribed, with adherence monitored.

Moxibustion Group: In addition to the control group’s medication regimen, moxibustion therapy was administered. Acupoints selected: Zusanli (bilateral), and Ashi points (at the 2–3 joints with most pronounced pain). Technique: Sparrow-peck moxibustion was applied to Zusanli and Ashi points for 10 minutes per point, three times weekly for 4 weeks.

No other disease−modifying antirheumatic drugs (DMARDs), biologics, or corticosteroids were permitted during the 4−week treatment period. Rescue medication (paracetamol, up to 2 g/day) was permitted for severe pain, and its use was recorded. Medication adherence was monitored by pill counts and patient diaries.

### 3.3 Moxibustion application method

Selection Criteria: Based on comprehensive analysis of acupoint selection principles documented in relevant literature, acupoint usage frequency (1, 25), and preliminary research findings from our team, Zusanli (bilateral) and Ashi points were ultimately selected as moxibustion sites. Among these, Ashi points were identified at 2–3 joints exhibiting the most pronounced pain.

Acupoint Localization: All acupoints were strictly localized according to the National Standard of the People’s Republic of China “Names and Locations of Acupoints” (GB/T 12346-2006).

**Figure 2:**
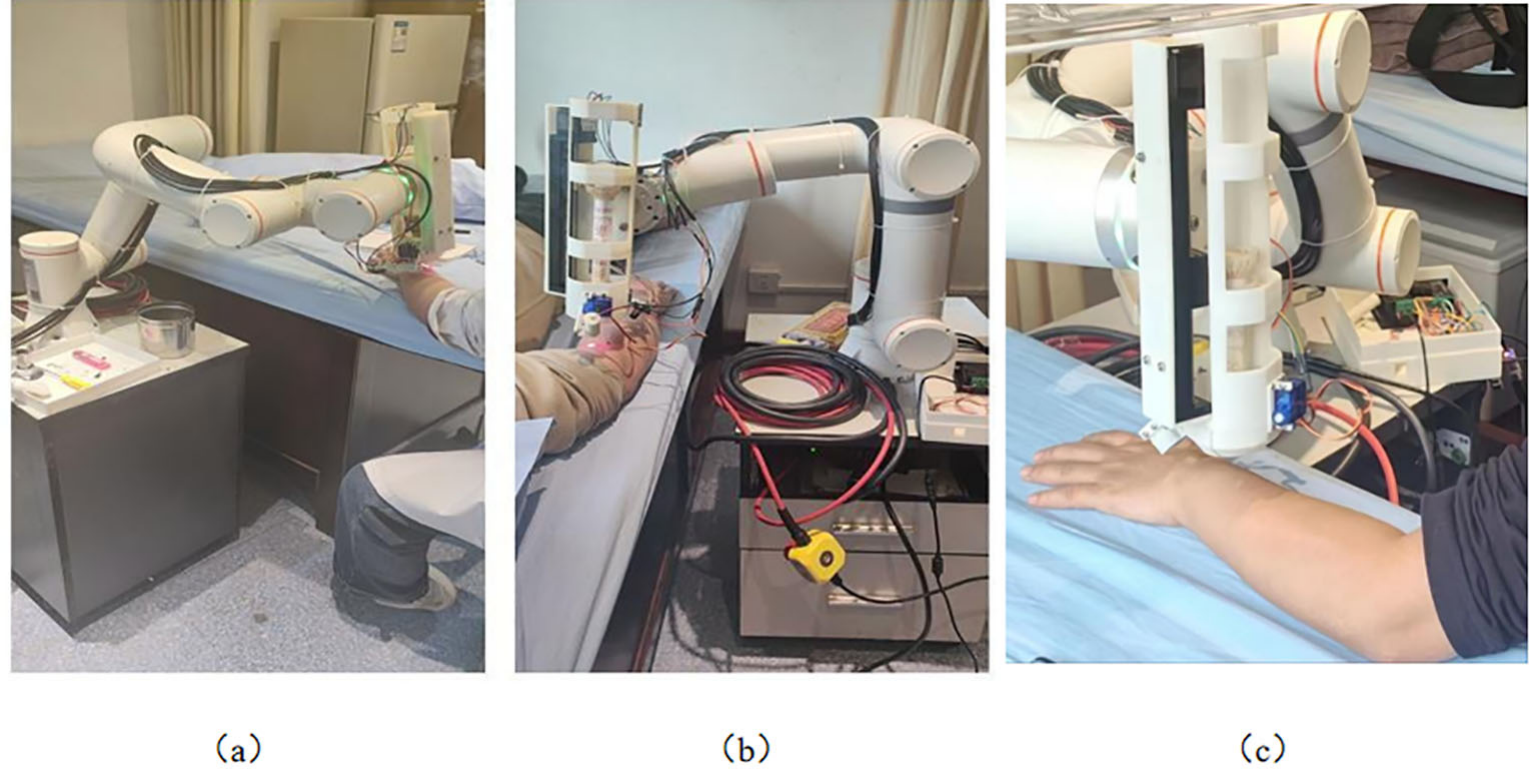
Moxibustion procedure and acupoint locations. **(a)** sparrow-peck moxibustion using the automated moxibustion arm; **(b)** Zusanli (ST36) acupoint on the lower leg; **(c)** Ashi point on a swollen metacarpophalangeal joint of the hand.

Operating Equipment and Procedure: The moxibustion group employed an automated moxibustion arm to deliver sparrow-peck moxibustion to the selected points. The device was jointly developed by Chengdu University of TCM and University of Electronic Science and Technology of China (Model: AJ-UESTC-V01; CE certificate No. M.2021.206.C63018). Key parameters include: working distance 3 cm, target temperature 43 ± 1 °C, and “bird-pecking” motion (40 cycles/min, 1 -3cm amplitude). The device was operated by two licensed acupuncturists (≥3 years of experience) after a 2-hour standardized training session. Daily calibration checks ensured consistency. This robotic arm fully executes the preset moxibustion treatment protocol. For detailed operational procedures and functional validation, refer to our group’s prior report”Functional Validation of the Automated Moxibustion Arm” (13).The specific method is as follows:

Sparrow-Peck Moxibustion (Zusanli, Ashi Point; using the finger’s small joint as an example):

The patient assumes a supine or seated position (refer to **Figures 2a–c**). Locate Zusanli (on the lateral aspect of the lower leg, 3 cun below the knee cap, one finger-width lateral to the anterior border of the tibia), and the proximal interphalangeal or metacarpophalangeal joints exhibiting swelling or pain. Set the moxibustion robotic arm’s movement parameters: Vertical direction: Frequency: 40 times/minute, Amplitude: 1–3 cm; Horizontal direction: Spiral progressive scanning covering the painful area. Secure the ignited end of the moxa stick to the moxibustion robotic arm. Instead of maintaining a fixed distance from the treatment site, move the arm up and down like a bird pecking, or move it evenly left and right, or rotate it repeatedly while applying moxibustion. Both practitioner and patient must maintain high concentration to prevent burns. If the patient experiences excessive skin heat, immediately remove the moxa cone. After a brief pause, resume treatment. Each site receives a total of 10 minutes of moxibustion, concluding when localized skin flushing is observed.

### 3.4 Observation indicators

Clinical Symptom Assessment Indicators: Visual Analog Scale (VAS), Morning Stiffness Score, 28-Joint Tenderness Count, 28-Joint Swelling Count, 28-Joint Disease Activity Score (DAS28),Health Assessment Questionnaire; Routine Clinical Laboratory Indicators: Core Serological Markers for Rheumatoid Arthritis (RF, ESR, CRP); Venous blood samples were collected before and after treatment. The following serum markers were detected using ELISA: Inflammatory cytokines (TNF-α, IL-17A) Wnt/β-catenin pathway molecules(wingless-type MMTV integration site family member 3A (WNT3A),low-density lipoprotein receptor-related protein 6 (LRP-6),glycogen synthase kinase-3 beta (GSK-3β), β-catenin). Bone metabolism markers [osteoprotegerin (OPG),beta-C-terminal telopeptide of type I collagen (β-CTX)].

### 3.5 Statistical methods

All data were analyzed using SPSS 26.0. Normality of continuous variables was assessed using the Shapiro–Wilk test. Continuous data are presented as mean ± standard deviation (Mean ± SD) for descriptive purposes, regardless of their distribution, to maintain uniformity across tables.

Primary analysis: Between-group comparisons (moxibustion group vs. control group) were performed using the Mann–Whitney U test on change scores (post-treatment minus pre-treatment), because the data were not normally distributed and baseline levels were well balanced between groups. Intragroup comparisons (pre- vs. post-treatment) were conducted using the Wilcoxon signed-rank test.

Sensitivity analysis: To adjust for potential baseline imbalances and other covariates, we performed multiple linear regression (ANCOVA) for each clinical outcome and serum biomarker. Each model included group (moxibustion vs. control), baseline value of the respective outcome, age, sex, and disease duration. The results for clinical outcomes are presented in **Supplementary Tables S1**–**S6** and summarized in **Table 2**; the results for serum biomarkers are presented in **Supplementary Tables S7**–**S14** and summarized in **Supplementary Table 15**.

**Table 2:** Adjusted treatment effects of moxibustion vs. control on clinical outcomes and serum β−catenin and β−CTX (multiple linear regression).

Outcome	Beta (95% CI)	*P* value
VAS score (points)	-1.755 (-2.406, -1.105)	< 0.001
Morning stiffness score (points)	-1.547 (-2.151, -0.943)	< 0.001
Tender joint count	-2.373 (-3.049, -1.698)	< 0.001
Swollen joint count	-2.173 (-2.797, -1.548)	< 0.001
DAS28 score	-0.710 (-0.994, -0.427)	< 0.001
HAQ score	-0.198 (-0.248, -0.147)	< 0.001
β-catenin	-34.621 (-55.41, -13.83)	0.002
β-CTX	-15.648 (-26.20, -5.10)	0.005

Beta, adjusted regression coefficient; CI, confidence interval. A negative beta indicates greater improvement in the moxibustion group compared with the control group. Each model was adjusted for baseline value, age, disease duration, and sex. Statistical significance was defined as P < 0.05.

Categorical variables were expressed as counts (percentages) and analyzed using the chi-square (χ²) test or Fisher’s exact test, as appropriate. For multiple comparisons, the Bonferroni correction was applied to adjust the significance level. All tests were two-sided, and a P value < 0.05 was considered statistically significant.

## 4 Result

### 4.1 Baseline comparability

There were no statistically significant differences between the two patient groups in terms of age, gender, disease duration, disease activity (DAS28), or baseline levels of all observed serum markers (*P* > 0.05), indicating comparability.

#### 4.1.1 Comparison of general information

Baseline demographic characteristics of patients in the moxibustion group and control group prior to treatment (see **Table 3**).

**Table 3:** Gender, age, and disease duration (mean ± SD) in two groups of RA patients.

Group	n	Gender/cases [n(%)]		Age/years	Duration of illness/years
		Male	Female		
Moxibustion Group	32	3 (9.4%)	29 (90.6%)	53.50 ± 10.81	6.13 ± 2.28
Control group	33	3 (9.1%)	30 (90.9%)	55.15 ± 10.11	5.91 ± 2.24
statistical value		–		t=0.637	t=-0.385
*P-*value		1.000		0.527	0.702

For intergroup comparisons, Fisher’s exact test was used for gender, while independent samples t-tests were employed for age and disease duration.

As shown in **Table 3**, there were no statistically significant differences between the two groups in terms of gender distribution, mean age, and mean disease duration (*P* > 0.05), indicating that the two groups of patients were balanced and comparable in demographic characteristics.

### 4.2 Comparison of clinical symptoms and functional scores

**Figure 3:**
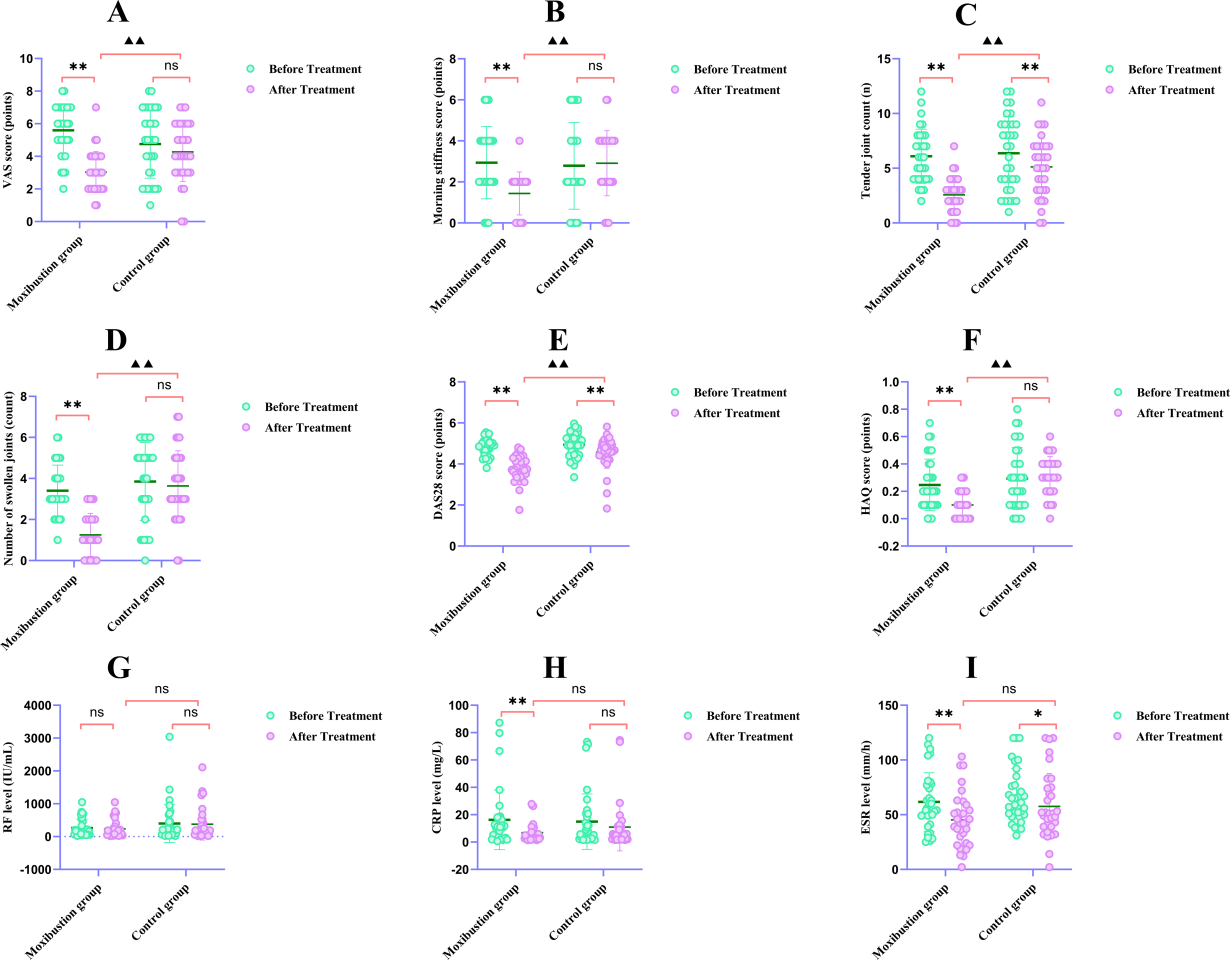
Clinical features and laboratory parameters before and after treatment. **(A–F)** Clinical symptoms: **(A)** VAS score, **(B)** morning stiffness score, **(C)** tender joint count, **(D)** swollen joint count, **(E)** DAS28 score, **(F)** HAQ score. **(G–I)** Laboratory parameters: **(G)** RF level, **(H)** CRP level, **(I)** ESR level. *Data are mean ± SD. For within-group comparisons: *P < 0.05, **P < 0.01 vs. pre‑treatment within the same group. For between-group comparisons of improvement values (post‑treatment minus pre‑treatment): ▲*P* < 0.05, ▲▲*P* < 0.01 vs. control group. ns, not significant.

Comparison of clinical symptoms and functional scores between the two patient groups before and after treatment (see **Table 4**; **Figures 3A–F**).

**Table 4:** Clinical symptoms and functional scores before and after treatment [mean ± SD, with median (IQR)].

Project	Group	Before treatment	After treatment	Z (*P*) within the group	Intergroup Z (*P*)
VAS Score (Points)	Moxibustion group (n=32)	5.59 ± 1.54 [6.00]	2.91 ± 1.06^**▴▴^ [3.00]	-4.89 (<0.001)	-4.48 (<0.001)
	Control group (n=33)	4.76 ± 2.09 [5.00]	4.27 ± 1.81 [4.00]	-1.61 (0.108)	
Morning stiffness score (points)	Moxibustion group (n=32)	2.94 ± 1.76 [2.00]	1.44 ± 1.05^**▴▴^ [1.00]	-3.97 (<0.001)	-3.36 (0.001)
	Control group (n=33)	2.79 ± 2.12 [2.00]	2.91 ± 1.59 [4.00]	-0.36 (0.717)	
Number of tender joints	Moxibustion group (n=32)	6.09 ± 2.48 [6.00]	2.56 ± 1.63^**▴▴^ [3.00]	-4.97 (<0.001)	-4.52 (<0.001)
	Control group (n=33)	6.36 ± 3.34 [7.00]	5.12 ± 2.70^**^ [5.00]	-3.46 (0.001)	
Number of swollen joints	Moxibustion group (n=32)	3.41 ± 1.24 [3.00]	1.25 ± 1.05^**▴▴^ [1.00]	-4.91 (<0.001)	-4.41 (<0.001)
	Control group (n=33)	3.85 ± 1.91 [5.00]	3.64 ± 1.24 [3.00]	-0.71 (0.480)	
DAS28 score (points)	Moxibustion group (n=32)	4.84 ± 0.42 [4.93]	3.76 ± 0.63^**▴▴^ [3.75]	-4.94 (<0.001)	-5.47 (<0.001)
	Control group (n=33)	4.94 ± 0.61 [4.91]	4.57 ± 0.78^**^ [4.67]	-3.46 (0.001)	
HAQ Score	Moxibustion group (n=32)	0.25 ± 0.19 [0.20]	0.10 ± 0.102^**▴▴^ [0.10]	-4.26 (<0.001)	-3.93 (<0.001)
	Control group (n=33)	0.29 ± 0.23 [0.30]	0.312 ± 0.141 [0.30]	-0.81 (0.419)	

Data are presented as mean ± SD with median [IQR] in brackets. All comparisons employed the Mann–Whitney U test for between−group comparisons, and Wilcoxon signed−rank test for within−group comparisons. For within-group comparisons, ^*^P < 0.05 indicates significance compared with pre-treatment values; ^**^P < 0.01 indicates a more significant difference. For between-group comparisons of improvement values (post-treatment minus pre-treatment), ^▴^*P* < 0.05 and ^▴▴^*P* < 0.01 indicate that the moxibustion group showed significantly greater improvement than the control group. For HAQ score, higher values indicate worse physical function.

As shown in **Table 4**; **Figures 3A–F**:

1. Intragroup comparison (changes within each group before vs. after treatment).

Moxibustion group: All six indicators (VAS score, morning stiffness score, number of tender joints, number of swollen joints, DAS28 score, and HAQ score) improved significantly after treatment compared with baseline (**P < 0.01 for all).

Control group: Only the number of tender joints and DAS28 score showed significant improvement after treatment (**P < 0.01), whereas VAS score, morning stiffness score, number of swollen joints, and HAQ score did not change significantly (P > 0.05).

2. Intergroup comparison (post-treatment differences between groups).

After treatment, the moxibustion group showed significantly greater improvement than the control group in all six indicators (^▴▴^*P* < 0.01 for morning stiffness, VAS, tender joints, swollen joints, DAS28, and HAQ).

#### 4.2.1 Conclusion

In RA patients, the moxibustion group demonstrated significantly better efficacy than the control group in improving morning stiffness, pain (VAS), tender/swollen joint counts, disease activity (DAS28), and physical function (HAQ). While the control group showed some improvement in tender joints and DAS28, the magnitude and breadth of improvement were markedly inferior to those observed in the moxibustion group.

### 4.3 Comparison of laboratory serological indicators

Comparison of conventional clinical laboratory indicators such as RF, CRP, and ESR between the two groups of RA patients before and after treatment (see **Table 5**; **Figures 3G–I**).

**Table 5:** Routine laboratory parameters before and after treatment [mean ± SD, median (IQR)].

Project	Time	Control group	Moxibustion group	*P*-value
		(n=33)	(n=32)	
RF(IU/mL)	Before treatment	399.26 ± 581.74 [203.00]	378.02 ± 485.78 [184.00]	0.369
	After treatment	261.88 ± 271.12 [166.00]	233.57 ± 271.61 [112.73]	
CRP(mg/L)	Before treatment	14.94 ± 20.34 [5.75]	16.24 ± 21.84 [10.55]	0.537
	After treatment	10.912 ± 17.324 [5.13]	6.802 ± 7.353** [3.55]	
ESR(mm/h)	Before treatment	66.88 ± 25.66 [61.00]	61.66 ± 26.66 [55.00]	0.379
	After treatment	57.545 ± 29.969* [51.00]	45.313 ± 24.662** [43.00]	

Data are presented as mean ± SD with median [IQR] in brackets. Due to non-normal distribution, statistical comparisons were performed using non-parametric tests: Mann–Whitney U test for between-group comparisons, and Wilcoxon signed-rank test for within-group comparisons. Intragroup comparisons: Compared with pre-treatment levels, ^*^P < 0.05, ^**^P < 0.01; Intergroup comparisons of improvement values (post-treatment minus pre-treatment): ^▴^P < 0.05, ^▴▴^P < 0.01 (moxibustion group vs. control group).

As shown in **Table 5**; **Figures 3G–I**: There were no statistically significant differences in routine clinical laboratory parameters between the two groups of RA patients before treatment (*P* > 0.05), indicating comparable baseline conditions.

Based on **Table 5** data and **Figures 3G–I**, the summary is as follows:

Intragroup Comparison:

Control Group: Only ESR levels showed a significant decrease compared to pre-treatment (^*^P < 0.05), while changes in CRP and RF were not statistically significant (*P*>0.05).

Moxibustion Group: Both ESR and CRP levels decreased significantly compared to pre-treatment (^**^P < 0.01), but changes in RF were not statistically significant (*P*>0.05).

Intergroup comparisons:

After treatment, intergroup comparisons of RF, CRP, and ESR levels in both groups showed no statistically significant differences (all *P* > 0.05). Thus, at the end of treatment, none of these conventional laboratory indicators differed significantly between the two groups.

### 4.4 Comparison of serum levels of OPG, IL-17A, β-catenin, LRP-6, GSK-3β, WNT3A, β-CTX, and TNF-α

**Figure 4:**
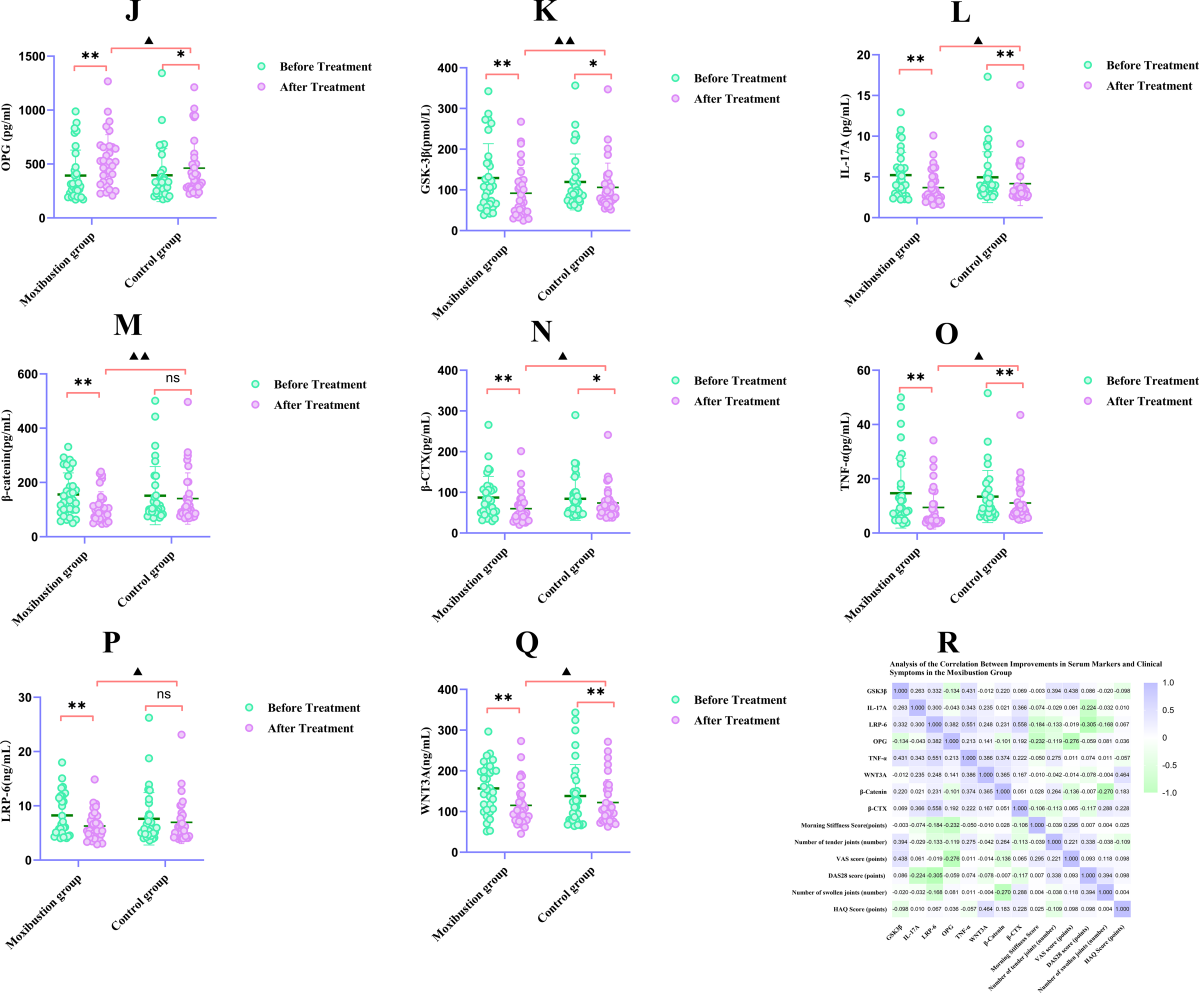
Serum biomarker levels and correlation analysis in RA patients. **(J–Q)** Serum biomarker levels: **(J)** OPG, **(K)** GSK-3β, **(L)** IL-17A, **(M)** β-catenin, **(N)** β-CTX, **(O)** TNF-α, **(P)** LRP-6, **(Q)** WNT3A. **(R)** Spearman's correlation matrix. *Data are mean ± SD. For within-group comparisons, *P < 0.05, **P < 0.01 vs. pre‑treatment within the same group; For between-group comparisons, ▲*P* < 0.05, ▲▲*P* < 0.01 vs. control group for improvement values (post‑treatment minus pre‑treatment). ns, not significant.

Comparison of serum levels of OPG, IL-17A, β-catenin, LRP-6, GSK-3β, WNT3A, β-CTX, and TNF-α in RA patients before and after treatment (see **Table 6**; **Figures 4J–Q**).

**Table 6:** Serum biomarker levels before and after treatment [mean ± SD, median (IQR)].

Testing criteria	Time	Control group (n=33)	Moxibustion group (n=32)	Statistical value	*P*-value
OPG (pg/mL)	Before treatment	395.88 ± 245.44 [330.54]	392.61 ± 236.29 [299.65]	Z = -0.446	0.656
	After treatment	462.16 ± 264.83^*^ [375.64]	528.75 ± 243.21^**▴^ [505.26]		
IL-17A (pg/mL)	Before treatment	4.96 ± 3.12 [3.89]	5.22 ± 2.80 [4.25]	Z = -0.564	0.573
	After treatment	4.17 ± 2.70^**^ [3.28]	3.69 ± 2.02^**▴▴^ [2.84]		
β-Catenin (ng/mL)	Before treatment	151.48 ± 107.39 [104.48]	155.57 ± 79.51 [134.78]	Z = -0.774	0.439
	After treatment	140.731 ± 94.287 [101.36]	109.276 ± 56.38^**▴▴^ [98.74]		
LRP-6 (ng/mL)	Before treatment	7.61 ± 4.82 [5.77]	8.23± 3.90 [6.34]	Z = -0.866	0.386
	After treatment	6.953 ± 3.914 [6.13]	6.279 ± 2.669^**▴^ [5.39]		
GSK-3β (pmol/L)	Before treatment	119.50± 68.68 [93.60]	129.08 ± 84.27 [104.61]	Z = -0.007	0.995
	After treatment	106.28 ± 59.69^*^ [91.80]	91.86 ± 62.94^**▴▴^ [71.45]		
WNT3A (ng/mL)	Before treatment	138.22 ± 77.37 [123.39]	156.89 ± 60.84 [155.93]	Z = -1.771	0.076
	After treatment	122.059 ± 56.344^**^ [104.22]	115.293 ± 52.212^**▴^ [100.61]		
β-CTX (ng/mL)	Before treatment	84.29 ± 53.10 [62.25]	87.27 ± 52.03 [76.38]	Z = -0.354	0.723
	After treatment	73.739 ± 40.07^*^ [65.51]	59.828 ± 38.968^**▴^ [47.22]		
TNF-α (pg/mL)	Before treatment	13.45 ± 9.62 [9.23]	14.68± 12.87 [8.72]	Z = -0.656	0.512
	After treatment	11.068 ± 7.471^**^ [8.32]	9.41 ± 7.936^**▴^ [5.23]		

Data are presented as mean ± SD with median [IQR] in brackets. Due to non-normal distribution, statistical comparisons were performed using non-parametric tests: Mann–Whitney U test for between-group comparisons (Z value and P-value shown), and Wilcoxon signed-rank test for within-group comparisons. Intragroup comparisons: Compared with pre-treatment levels, ^*^P < 0.05, ^**^P < 0.01; Intergroup comparisons of improvement values (post-treatment minus pre-treatment): ^▴^*P* < 0.05, ^▴▴^*P* < 0.01 (moxibustion group vs. control group).

#### 4.4.1 Intragroup comparisons (pre- vs. post-treatment)

Control group: After treatment, serum levels of IL-17A, WNT3a, and TNF-α decreased significantly compared to pre-treatment (all *P* < 0.01); levels of OPG, GSK-3β, and β-CTX improved significantly compared to pre-treatment (all *P* < 0.05); while changes in β-Catenin and LRP-6 levels were not statistically significant (both *P*>0.05).

Moxibustion Group: After treatment, all observed serum markers (OPG, IL-17A, β-Catenin, LRP-6, GSK-3β, WNT3a, β-CTX, TNF-α) showed highly significant improvement compared to pre-treatment levels (all *P* < 0.01).

#### 4.4.2 Intergroup comparison (post-treatment)

Post-treatment intergroup comparison revealed that the moxibustion group demonstrated significantly greater improvement in multiple indicators compared to the control group: Serum levels of OPG and LRP-6 in the moxibustion group were significantly higher than those in the control group (both *P* < 0.05). Serum levels of IL-17A, β-Catenin, GSK-3β, WNT3a, β-CTX, and TNF-α in the moxibustion group were significantly superior to those in the control group (all *P* < 0.01).

### 4.5 Correlation analysis

#### 4.5.1 In the moxibustion group, there was a significant correlation between the changes in serum indicators before and after treatment

**Figure 5:**
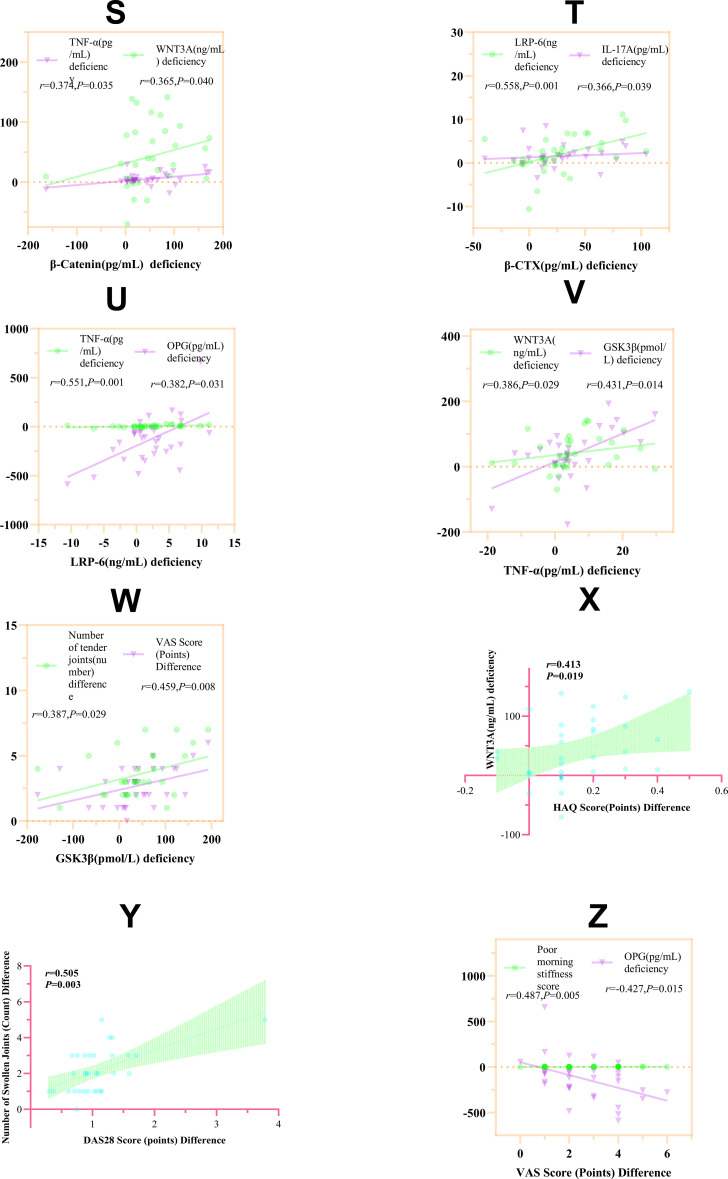
Correlation analysis of serum markers and clinical symptoms in the moxibustion group. **(S–Z)** Spearman's correlation analyses: **(S)** β-catenin vs. TNF-α and WNT3A; **(T)** β-CTX vs. LRP-6 and IL-17A; **(U)** LRP-6 vs. TNF-α and OPG; **(V)** TNF-α vs. WNT3A and GSK-3β; **(W)** GSK-3β vs. VAS score and tender joint count; **(X)** WNT3A vs. HAQ score; **(Y)** DAS28 score vs. swollen joint count; **(Z)** VAS score vs.OPG and morning stiffness score. *r* > 0 indicates a positive correlation, *r* < 0 indicates a negative correlation. *P* < 0.05 indicates a statistically significant correlation.

As shown in **Table 7** and **Figure 4R**, there was a significant correlation between the changes in serum indicators before and after treatment. The difference in β-catenin levels before and after treatment in the RA group was significantly correlated with the differences in TNF-α and WNT3A levels (*P* < 0.05), and all correlations were positive (*r* > 0),See **Figure 5S**. Correlation analysis revealed positive associations between serum indicators and bone resorption marker β-CTX. Specifically, LRP-6 and IL-17A levels were significantly correlated with β-CTX levels (*r* > 0, *P* < 0.01; *r* > 0, *P* < 0.05),See **Figure 5T**. Significant positive correlations were observed between LRP-6 and both TNF-α (*r* > 0, *P* < 0.01) and OPG (*r* > 0, *P* < 0.05),See **Figure 5U**. Significant positive correlations were also found between TNF-α and WNT3A (*r* > 0, *P* < 0.05), as well as between GSK3β and TNF-α (*r* > 0, *P* < 0.05),See **Figure 5V**. In summary, the observed correlations imply that TNF-α and WNT3A converge to activate the canonical Wnt/β-catenin signaling pathway, central to which is the accumulation of β-catenin.

**Table 7:** Correlation among improvement values of serum markers in the moxibustion group.

Indicator A	Indicator B	Correlation coefficient (r)	*P*-value
LRP-6	TNF-α	0.551	0.001**
LRP-6	β-CTX	0.558	0.001**
IL-17A	β-CTX	0.366	0.039*
TNF-α	WNT3A	0.386	0.029*
TNF-α	β-Catenin	0.374	0.035*
WNT3A	β-Catenin	0.365	0.040*
GSK3β	TNF-α	0.431	0.014*
OPG	LRP-6	0.382	0.031*

^*^P < 0.05, ^**^P < 0.01.

#### 4.5.2 Correlation analysis between serum-related indicators and clinical symptoms before and after moxibustion treatment

**Table 8** presents the correlation analysis between serum marker improvement and clinical symptom improvement in the moxibustion group.

**Table 8:** Correlation analysis between serum marker improvement and clinical symptom improvement in the moxibustion group.

Grouping	Clinical symptom indicators	Serum markers	Correlation coefficient (r)	*P-*value
Function and Molecules	HAQ Score	WNT3A	0.413	0.019*
Pain and Molecules	VAS Score (Pain)	GSK3β	0.459	0.008**
	Number of tender joints	GSK3β	0.387	0.029*
	VAS Score (Pain)	OPG	-0.427	0.015*
Correlation between symptoms	VAS Score (Pain)	Morning Stiffness Score	0.487	0.005**
Correlation among clinical indicators	DAS28 score	Number of swollen joints	0.505	0.003**

^*^P < 0.05, ^**^P < 0.01.

Positive correlation: Decreased serum GSK-3β levels were positively correlated with reduced joint tenderness counts (*r* > 0, *P* < 0.05) and pain VAS scores (*r* > 0, *P* < 0.01), See **Figure 5W**; increased WNT3A levels were positively correlated with improved HAQ functional scores (*r* > 0, *P* < 0.05), See **Figure 5X**. Furthermore, strong correlations existed within clinical symptoms: improvement in pain VAS scores positively correlated with improvement in morning stiffness scores (*r* > 0, *P* < 0.01), and improvement in DAS28 disease activity scores positively correlated with reduced joint swelling counts (*r* > 0, *P* < 0.01), See **Figure 5Y**.

Negative correlations: Elevated serum OPG levels were negatively correlated with reduced pain VAS scores (*r* < 0, *P* < 0.05), See **Figure 5Z**.

### 4.6 Multiple linear regression analysis (sensitivity analysis)

To further adjust for potential baseline imbalances, we performed multiple linear regression (ANCOVA) as a sensitivity analysis for all clinical outcomes and serum biomarkers. Each model included the following independent variables: group (moxibustion vs. control), baseline value of the respective outcome, age, disease duration, and sex.

#### 4.6.1 Clinical outcomes

**Figure 6:**
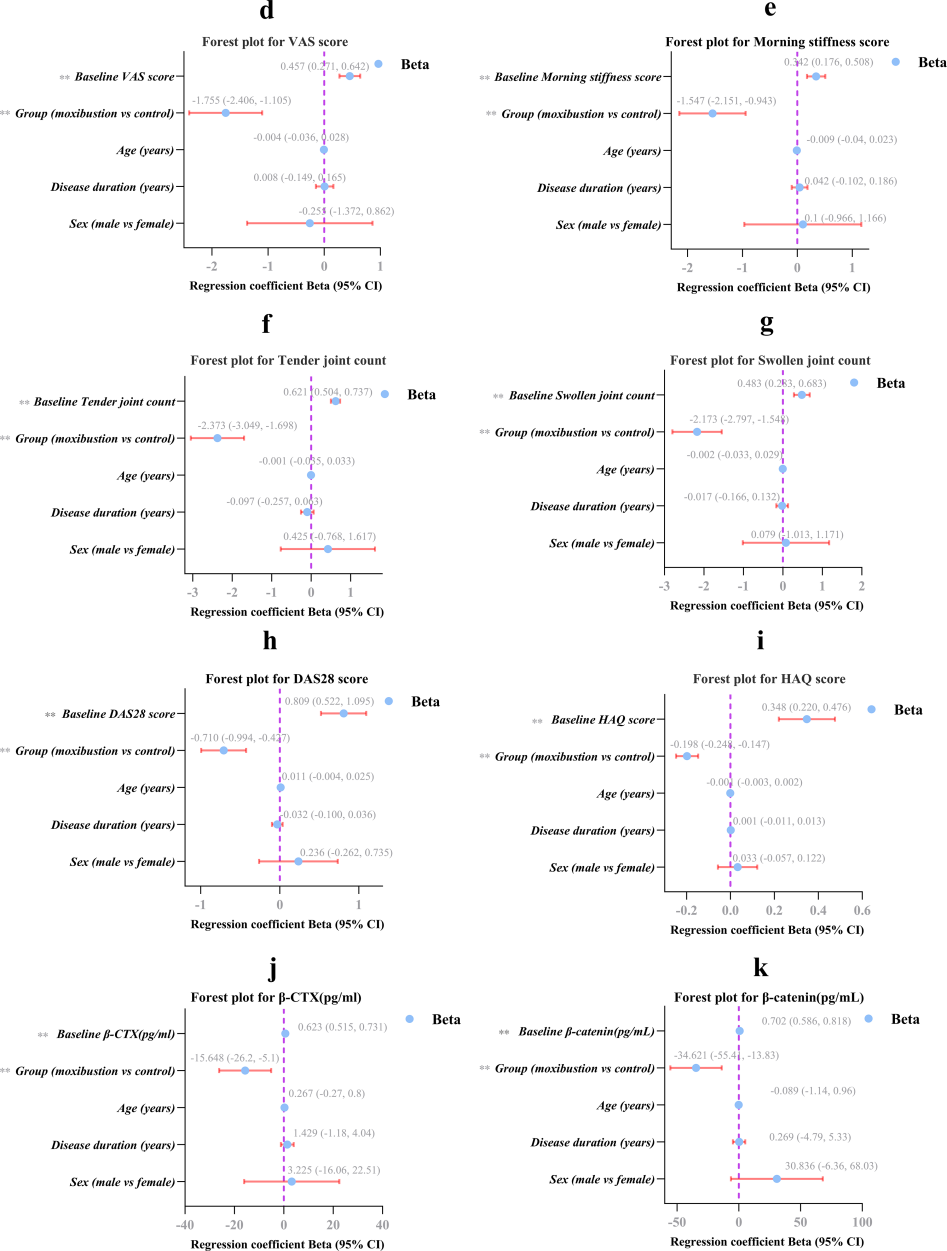
Forest plot of multiple linear regression analysis **(d–k)**. Panels **(d–i)** present forest plots for VAS, morning stiffness, tender joint count, swollen joint count, DAS28, and HAQ, respectively. **(j)** β-CTX, **(k)** β-catenin.Forest plots showing the adjusted regression coefficients (beta) and 95% confidence intervals (CIs) for all predictors across the clinical outcomes **(d–i)** and serum biomarkers **(j–k).** For each outcome, a separate multiple linear regression model was used with baseline value, group, age, disease duration, and sex as covariates. The vertical dashed line indicates beta = 0 (no effect). Points to the left of the line indicate improvement with moxibustion (for group variable) or with lower baseline scores (for baseline variable). **< 0.01 indicates statistical significance. CI, confidence interval.

The adjusted treatment effects for the six clinical outcomes (VAS, morning stiffness, tender joint count, swollen joint count, DAS28, and HAQ) are summarized in **Table 2**. After adjusting for covariates, the moxibustion group remained significantly superior to the control group for all six outcomes (all P < 0.001). **Figure 6** (panels d−i) presents forest plots showing the adjusted regression coefficients (β) and 95% confidence intervals (CIs) for each predictor across these outcomes. For the group variable, all β estimates were negative with CIs entirely below zero, confirming the robust clinical efficacy of moxibustion.

#### 4.6.2 Serum biomarkers

The same regression models were applied to each serum biomarker (OPG, IL−17A, β−catenin, GSK−3β, β−CTX, TNF−α, LRP−6, and WNT3A). Full results (including coefficients for all covariates) are provided in **Supplementary Table 7-S14**. A summary of the adjusted group effects is shown in **Supplementary Table 15**. After adjustment, only β−catenin (β = –34.62, 95% CI: –55.41 to –13.83, P = 0.002) and β−CTX (β = –15.65, 95% CI: –26.20 to –5.10, P = 0.005) remained statistically significant. The other six biomarkers showed no significant group differences (all P > 0.05).

The forest plot (**Figures 6d–k**) visually presents the adjusted regression coefficients and 95% CIs after adjusting for baseline values, age, disease duration, and sex (all *P* < 0.01).

## 5 Discussion

### 5.1 Clinical efficacy analysis

The combination therapy of moxibustion significantly improved RA symptoms, with functional recovery closely associated with improved bone metabolism indicators (14). Our findings indicate that moxibustion combined with conventional Western medication outperformed Western medication alone in alleviating joint tenderness, swelling, morning stiffness, disease activity (DAS28), and physical function (HAQ) in RA patients. The difference was particularly significant in relieving morning stiffness and improving HAQ scores. This finding aligns with the conclusions of most clinical studies indicating that moxibustion or acupuncture as adjunctive therapy for RA can effectively alleviate joint symptoms and enhance quality of life (15, 16).

Notably, this study identified an association between clinical symptom improvement and bone metabolic regulation. Compared to previous research primarily focusing on moxibustion’s anti-inflammatory and analgesic effects (17, 18), this study further reveals its potential value in promoting bone metabolic balance. Correlation analysis showed that elevated serum osteoprotegerin (OPG) levels, representing bone protection, were negatively correlated with improved pain (VAS) scores, while improved pathway ligand WNT3A levels were positively correlated with improved HAQ functional scores. This suggests that the clinical efficacy of moxibustion, particularly in pain relief and functional recovery, may partly stem from its positive regulation of bone metabolic balance and the downstream Wnt/β-catenin pathway, rather than solely from anti-inflammatory effects. We propose that moxibustion may improve local microcirculation and the cellular environment through its “warming and unblocking” action, thereby creating conditions conducive to bone repair. This process likely influences bone remodeling while alleviating symptoms.

### 5.2 Investigation of serum marker mechanisms

Moxibustion Multi-Target Regulation of the “Inflammation-Pathway-Bone Metabolism” Network This study systematically elucidated the mechanism of moxibustion by detecting three types of serum biomarkers. The results and discussion are as follows:

(1) Inflammatory cytokines (TNF-α, IL-17A): Moxibustion Effectively Suppresses Key Pro-inflammatory Factors: The results of this study indicate that combined moxibustion therapy significantly reduces serum levels of TNF-α and IL-17A. This finding aligns with extensive literature confirming the anti-inflammatory effects of moxibustion/acupuncture (19, 20). TNF-α and IL-17A are core cytokines driving synovitis and bone destruction in RA, with TNF-α known to induce the Wnt pathway inhibitor Dickkopf-1 (DKK-1), impairing osteoblast differentiation (21, 40). Our findings are consistent with Tao et al. (2023) showing moxibustion reduces β-catenin and TNF-α in RA patients (29).A recent mechanistic study (Lai et al., 2025) identified that moxibustion-derived compounds directly bind TNF-α, supporting our observed reduction (30).Unlike studies focusing solely on their direct pro-inflammatory effects, this research examines them within the “inflammation-bone metabolism” interactive network. Moxibustion downregulates both factors, not only alleviating inflammation but potentially indirectly relieving its suppression of the Wnt/β-catenin pathway, thereby creating prerequisites for subsequent bone formation regulation.
(2) Wnt/β-catenin pathway proteins (WNT3A, LRP-6, GSK-3β, β-catenin): Moxibustion regulates pathway homeostasis: Studies reveal that following moxibustion treatment, beneficial changes occur in serum levels of WNT3A, LRP-6, and β-catenin, while GSK-3β activity is suppressed. In bone biology, the Wnt/β-catenin pathway serves as the primary regulator of osteogenic differentiation, with its activation promoting bone formation (22, 41). Unlike animal studies where moxibustion activates Wnt/β-catenin in bone during fracture healing (31), our clinical data show downregulation of the pathway, reflecting context-dependent regulation (RA synovium vs. osteoblasts).Unlike some studies reporting “moxibustion activates the Wnt pathway” in osteoporosis models (22, 23), this research observed “downregulation” of abnormally elevated markers and “inhibition” of negative regulatory factors in RA patients. The bidirectional regulation of the Wnt/β-catenin pathway by moxibustion is context-dependent. In RA, chronic inflammation drives aberrant activation of the Wnt/β-catenin pathway in synovial fibroblasts (32, 33); the acidic microenvironment further enhances RASF proliferation via ASIC1a/Wnt/β-catenin signaling (34). Moxibustion reduces inflammatory cytokines, thereby indirectly suppressing this pathological overactivation—a normalization toward baseline levels. In contrast, in osteoporosis where the pathway is downregulated in osteoblasts, moxibustion enhances its activity to promote bone formation (35). This homeostatic regulation aligns with the multi-target, multi-level actions of acupuncture and moxibustion (36, 37).
(3) Bone Metabolic Markers (OPG, β-CTX): Moxibustion Promotes Bone Protection and Inhibits Bone Resorption: The most direct finding in mechanism studies is that moxibustion significantly elevates OPG levels while reducing β-CTX levels. OPG is a key osteoprotective factor that inhibits osteoclast generation by blocking RANKL signaling (24); β-CTX is a specific marker of bone resorption. These findings align with reports showing moxibustion or electroacupuncture improves bone metabolism indicators in postmenopausal osteoporosis studies (25, 26). The novelty of this research lies in demonstrating that moxibustion exerts similar regulatory effects in RA, a disease characterized by inflammatory bone destruction. Combined with correlation analyses (e.g., β-catenin changes positively correlated with TNF-α and WNT3A changes), we propose that moxibustion may exert its dual effect—increasing OPG (promoting bone protection) and decreasing β-CTX (inhibiting bone resorption)—through a synergistic combination of the aforementioned anti-inflammatory actions and regulation of Wnt pathway homeostasis. This directly targets the core pathological mechanism of RA: inflammatory bone destruction.

### 5.3 Consistency and discrepancy between primary and sensitivity analyses: anti-inflammatory effects and bone-related markers

For clinical outcomes, both the primary change-score analysis and the sensitivity analysis (ANCOVA) demonstrated consistently significant improvements in the moxibustion group, confirming the clinical efficacy of moxibustion.

For serum biomarkers, the primary change-score analysis (Mann-Whitney U) showed that moxibustion significantly reduced the levels of the pro-inflammatory cytokines IL-17A and TNF-α compared with the control group (both P < 0.05, **Table 6**). This finding supports the anti-inflammatory effect of moxibustion in RA patients. In the sensitivity analysis using ANCOVA, which adjusts for minor baseline numerical differences, the group differences for IL-17A and TNF-α were in the same direction (negative) but did not reach statistical significance (both P = 0.107). This discrepancy is not unexpected. Although baseline levels were well balanced between groups (all P > 0.05), there were numerical differences that were adjusted for in the ANCOVA. Given that our data were not normally distributed and the non-parametric change-score analysis is robust to outliers, we consider the primary analysis valid. The fact that both analytical methods consistently showed significant reductions in β-catenin and β-CTX (bone-related markers) further supports the multi-target benefits of moxibustion. Larger studies are warranted to confirm the anti-inflammatory effects of moxibustion using more stringent adjustment methods.

### 5.4 Research significance and limitations

The significance of this study lies not only in validating the clinical efficacy of moxibustion therapy for RA but also in preliminarily elucidating its multi-targeted, holistic regulatory characteristics from the perspective of the “inflammation-signaling pathway-bone metabolism” interactive network (27, 28). Limitations primarily include a limited sample size, lack of blinding for patients and practitioners, a relatively short observation period, and the absence of local tissue evidence. Future research should be refined through larger sample sizes, extended observation periods, and integration with basic experimental studies. This study has several limitations. First, the intervention duration was only 4 weeks, which is relatively short for a chronic disease such as RA. Stable changes in bone metabolism markers typically require longer observation periods; therefore, the changes in β−CTX observed in this study should be interpreted as early trends. Longer follow−up (e.g., 12 weeks or more) is needed to confirm sustained bone−protective effects. Second, we did not assess structural changes by imaging (e.g., X−ray or MRI), so the conclusion regarding prevention of joint damage remains preliminary. Future studies should extend the treatment and follow−up periods and include imaging endpoints to provide more definitive evidence. A limitation is that the primary change-score analysis and ANCOVA yielded different significance levels for the anti-inflammatory cytokines (IL-17A, TNF-α), likely due to minor baseline numerical differences. However, clinical outcomes were consistently significant in both analyses. Larger studies are needed to confirm the direct anti-inflammatory effects of moxibustion independent of baseline levels.

In summary, the combination of moxibustion and conventional Western medication for treating rheumatoid arthritis (RA) exerts synergistic effects in alleviating clinical symptoms and inhibiting inflammatory bone destruction through multiple target mechanisms. These include suppressing the TNF-α/IL-17A inflammatory axis, regulating the Wnt/β-catenin pathway homeostasis, enhancing OPG, and reducing β-CTX. This approach embodies the therapeutic principle of addressing both the symptoms and root causes of the disease.

## Data Availability

The raw data supporting the conclusions of this article will be made available by the authors, without undue reservation.
