# Supplementary material for: Mechanistic study on the reduction of TNF-α and β-CTX levels in RA patients by moxibustion combined with western medication through regulation of the Wnt/β-catenin pathway
Source: Front Immunol. 2026 May 22;17:1808931. doi: 10.3389/fimmu.2026.1808931 (PMC13236493; doi:10.3389/fimmu.2026.1808931)
Supplement: Supplementary file 1 [file DataSheet1.pdf]

Supplementary Table S1 Complete multiple linear regression results for VAS score, including sex, disease duration, age, group, and baseline VAS score as covariates.

Variable	Beta	Lower 95% CI	Upper 95% CI	*P* Value
Sex (male vs female)	-0.255	-1.372	0.862	0.649
Disease duration (years)	0.008	-0.149	0.165	0.922
Age (years)	-0.004	-0.036	0.028	0.812
Group (moxibustion vs control)	-1.755	-2.406	-1.105	**< 0.001**
Baseline VAS score	0.457	0.271	0.642	**< 0.001**

Note:Data are regression coefficients (beta) with 95% confidence intervals (CI) from multiple linear regression. Each model included sex, disease duration, age, group (moxibustion vs. control), and the respective baseline score as independent variables.P< 0.001indicates statistical significance.

Supplementary Table S2 Complete multiple linear regression results for morning stiffness score, including sex, disease duration, age, group, and baseline morning stiffness score as covariates.

Variable	Beta	Lower 95% CI	Upper 95% CI	*P* Value
Sex (male vs female)	0.1	-0.966	1.166	0.851
Disease duration (years)	0.042	-0.102	0.186	0.563
Age (years)	-0.009	-0.04	0.023	0.573
Group (moxibustion vs control)	-1.547	-2.151	-0.943	**< 0.001**
Baseline Morning stiffness score	0.342	0.176	0.508	**< 0.001**

Note: See Note under Supplementary Table S1. The baseline covariate corresponds to the respective outcome .

Supplementary Table S3 Complete multiple linear regression results for tender joint count, including sex, disease duration, age, group, and baseline tender joint count as covariates.

Variable	Beta	Lower 95% CI	Upper 95% CI	*P* Value
Sex (male vs female)	0.425	-0.768	1.617	0.479
Disease duration (years)	-0.097	-0.257	0.063	0.231
Age (years)	-0.001	-0.035	0.033	0.952
Group (moxibustion vs control)	-2.373	-3.049	-1.698	**< 0.001**
Baseline Tender joint count	0.621	0.504	0.737	**< 0.001**

Note: See Note under Supplementary Table S1. The baseline covariate corresponds to the respective outcome .

Supplementary Table S4 Complete multiple linear regression results for swollen joint count, including sex, disease duration, age, group, and baseline swollen joint count as covariates.

Variable	Beta	Lower 95% CI	Upper 95% CI	*P* Value
Sex (male vs female)	0.079	-1.013	1.171	0.885
Disease duration (years)	-0.017	-0.166	0.132	0.816
Age (years)	-0.002	-0.033	0.029	0.905
Group (moxibustion vs control)	-2.173	-2.797	-1.548	**< 0.001**
Baseline Swollen joint count	0.483	0.283	0.683	**< 0.001**

Note: See Note under Supplementary Table S1. The baseline covariate corresponds to the respective outcome .

Supplementary Table S5 Complete multiple linear regression results for DAS28 score, including sex, disease duration, age, group, and baseline DAS28 score as covariates.

Variable	Beta	Lower 95% CI	Upper 95% CI	*P* Value
Sex (male vs female)	0.236	-0.262	0.735	0.347
Disease duration (years)	-0.032	-0.1	0.036	0.349
Age (years)	0.011	-0.004	0.025	0.139
Group (moxibustion vs control)	-0.71	-0.994	-0.427	< **0.001**
Baseline DAS28 score	0.809	0.522	1.095	< **0.001**

Note: See Note under Supplementary Table S1. The baseline covariate corresponds to the respective outcome .

Supplementary Table S6 Complete multiple linear regression results for HAQ score, including sex, disease duration, age, group, and baseline HAQ score as covariates.

Variable	Beta	Lower 95% CI	Upper 95% CI	*P* Value
Sex (male vs female)	0.033	-0.057	0.122	0.47
Disease duration (years)	0.001	-0.011	0.013	0.898
Age (years)	-0.001	-0.003	0.002	0.441
Group (moxibustion vs control)	-0.198	-0.248	-0.147	< **0.001**
Baseline HAQ score	0.348	0.22	0.476	< **0.001**

Note: See Note under Supplementary Table S1. The baseline covariate corresponds to the respective outcome .

Supplementary Table S7. Complete multiple linear regression results for OPG level after treatment, including sex, disease duration, age, group, and baseline OPG score as covariates.

Variable	Beta	Lower 95% CI	Upper 95% CI	*P* Value
Sex (male vs female)	97.636	-69.93	265.20	0.258
Disease duration (years)	-7.589	-29.97	14.79	0.509
Age (years)	0.764	-3.96	5.49	0.753
Group (moxibustion vs control)	71.623	-22.23	165.48	0.140
Baseline OPG score	0.736	0.536	0.936	**< 0.001**

Note: See Note under Supplementary Table S1. The baseline covariate corresponds to the respective outcome.

Supplementary Table S8. Complete multiple linear regression results for GSK-3β level after treatment, including sex, disease duration, age, group, and baseline GSK-3β as covariates.

Variable	Beta	Lower 95% CI	Upper 95% CI	*P* Value
Sex (male vs female)	23.842	-17.78	65.46	0.266
Disease duration (years)	2.392	-3.33	8.11	0.416
Age (years)	0.111	-1.06	1.28	0.853
Group (moxibustion vs control)	-19.598	-42.91	3.71	0.105
Baseline GSK-3β	0.498	0.337	0.659	**< 0.001**

Note: See Note under Supplementary Table S1. The baseline covariate corresponds to the respective outcome.

Supplementary Table S9. Complete multiple linear regression results for IL-17A level after treatment, including sex, disease duration, age, group, and baseline IL-17A as covariates.

Variable	Beta	Lower 95% CI	Upper 95% CI	*P* Value
Sex (male vs female)	-1.575	-2.943	-0.207	**0.028**
Disease duration (years)	0.102	-0.086	0.290	0.292
Age (years)	0.006	-0.033	0.045	0.773
Group (moxibustion vs control)	-0.647	-1.423	0.129	0.107
Baseline IL-17A	0.586	0.449	0.723	**< 0.001**

Note: See Note under Supplementary Table S1. The baseline covariate corresponds to the respective outcome.

Supplementary Table S10. Complete multiple linear regression results for β-catenin level after treatment, including sex, disease duration, age, group, and baseline β-catenin as covariates.

Variable	Beta	Lower 95% CI	Upper 95% CI	*P* Value
Sex (male vs female)	30.836	-6.36	68.03	0.110
Disease duration (years)	0.269	-4.79	5.33	0.918
Age (years)	-0.089	-1.14	0.96	0.869
Group (moxibustion vs control)	-34.621	-55.41	-13.83	**0.002**
Baseline β-catenin	0.702	0.586	0.818	**< 0.001**

Note: See Note under Supplementary Table S1. The baseline covariate corresponds to the respective outcome.

Supplementary Table S11. Complete multiple linear regression results for β-CTX level after treatment, including sex, disease duration, age, group, and baseline β-CTX as covariates.

Variable	Beta	Lower 95% CI	Upper 95% CI	*P* Value
Sex (male vs female)	3.225	-16.06	22.51	0.744
Disease duration (years)	1.429	-1.18	4.04	0.287
Age (years)	0.267	-0.27	0.80	0.331
Group (moxibustion vs control)	-15.648	-26.20	-5.10	0.005
Baseline β-CTX	0.623	0.515	0.731	**< 0.001**

Note:See Note under Supplementary Table S1.

Supplementary Table S12. Complete multiple linear regression results for TNF-α level after treatment, including sex, disease duration, age, group, and baseline TNF-α as covariates.

Variable	Beta	Lower 95% CI	Upper 95% CI	*P* Value
Sex (male vs female)	-0.853	-5.65	3.94	0.729
Disease duration (years)	-0.015	-0.67	0.64	0.965
Age (years)	0.007	-0.13	0.14	0.916
Group (moxibustion vs control)	-2.245	-4.94	0.45	0.107
Baseline TNF-α	0.489	0.364	0.614	**< 0.001**

Note:See Note under Supplementary Table S1.

Supplementary Table S13. Complete multiple linear regression results for LRP- 6 level after treatment, including sex, disease duration, age, group, and baseline LRP- 6 as covariates.

Variable	Beta	Lower 95% CI	Upper 95% CI	*P* Value
Sex (male vs female)	-0.765	-3.12	1.59	0.528
Disease duration (years)	-0.103	-0.42	0.22	0.534
Age (years)	0.026	-0.04	0.09	0.448
Group (moxibustion vs control)	-0.903	-2.22	0.41	0.183
Baseline LRP- 6	0.485	0.326	0.644	**< 0.001**

Note:See Note under Supplementary Table S1.

Supplementary Table S14. Complete multiple linear regression results for WNT3A level after treatment, including sex, disease duration, age, group, and baseline WNT3A as covariates.

Variable	Beta	Lower 95% CI	Upper 95% CI	*P* Value
Sex (male vs female)	-11.687	-42.20	18.83	0.456
Disease duration (years)	-0.153	-4.26	3.95	0.942
Age (years)	0.434	-0.42	1.29	0.326
Group (moxibustion vs control)	-17.151	-34.35	0.05	0.055
Baseline WNT3A	0.598	0.471	0.725	**< 0.001**

Note:See Note under Supplementary Table S1.

Supplementary Table S15. Adjusted treatment effects of moxibustion vs. control on serum biomarkers (multiple linear regression)

Biomarker	*Beta (95% CI)*	*P value*
OPG	71.623 (-22.23, 165.48)	0.140
GSK-3β	-19.598 (-42.91, 3.71)	0.105
IL-17A	-0.647 (-1.423, 0.129)	0.107
β- catenin	-34.621 (-55.41, -13.83)	**0.002**
β- CTX	-15.648 (-26.20, -5.10)	**0.005**
TNF- α	-2.245 (-4.94, 0.45)	0.107
LRP- 6	-0.903 (-2.22, 0.41)	0.183
WNT3A	-17.151 (-34.35, 0.05)	0.055

Note: Beta= adjusted regression coefficient; CI = confidence interval. A negative β indicates greater reduction (improvement) in the moxibustion group compared with the control group. Each biomarker was analyzed in a separate multiple linear regression model that included the following covariates:group, baseline value of the respective biomarker, age, sex, and disease duration. Full regression results (including coefficients for all covariates) are presented in Supplementary Tables S7–S14. Bold P values indicate statistical significance ($P < 0.05$).
